# Decreasing Hemoglobin A1c: Progress or Pathology?

**DOI:** 10.7759/cureus.108194

**Published:** 2026-05-03

**Authors:** Daniel L Gehlbach, Chai L Arnold, Gerardo Moreno

**Affiliations:** 1 Family Medicine, David Geffen School of Medicine at University of California, Los Angeles (UCLA), Los Angeles, USA

**Keywords:** continuity of patient care, family medicine residency, hypoglycemia workup, pancreatic insulinoma, residency clinic, type 2 diabetes

## Abstract

A 67-year-old woman with multiple comorbidities presented to the clinic for a routine follow-up of her chronic conditions, including diabetes management. She had been followed by multiple residents over the years and was noted to have a declining hemoglobin A1c (HgbA1c) despite discontinuation of diabetes medications and no significant lifestyle changes.

The patient reported episodes of hypoglycemia, dizziness, and multiple falls, resulting in several emergency department visits. Her HgbA1c had decreased from a prior high of 10% to 5.4%. Further evaluation included laboratory tests, imaging, and an e-consult to Endocrinology. Diagnostic evaluation revealed elevated insulin (47.8 μU/mL; reference range: 2-25 μU/mL) and c-peptide (11.2 ng/mL; reference range: 0.5-2 ng/mL) in the presence of hypoglycemia (serum glucose 42 mg/dL). Computed tomography (CT) and magnetic resonance imaging (MRI) identified a mass in the pancreatic tail. Endocrinology recommended urgent surgical consultation, and one month later, the patient underwent distal pancreatectomy and splenectomy. Final pathology confirmed an insulinoma. She was subsequently followed by her primary care physician, surgical team, and Endocrinology, resumed metformin, and has had no further hypoglycemic episodes or falls.

Insulinoma is a rare diagnosis that, if overlooked, can lead to significant morbidity. While treatment is typically straightforward once identified, diagnosis often requires careful attention to subtle clinical clues. This case illustrates how a patient with multiple chronic conditions and an improving HgbA1c could easily be overlooked in a busy resident-run clinic. It also demonstrates the importance of careful evaluation before attributing improvements in HgbA1c solely to treatment adjustments, as well as the value of multidisciplinary coordination in diagnosing and treating underlying disease in a timely manner.

## Introduction

Resident physicians in primary care encounter unique clinical challenges during their training, including accurate diagnostic evaluations, formulating treatment plans for multiple issues, and establishing rapport with patients they may meet for the first time. While chronic conditions like type 2 diabetes, hypertension, and gastroesophageal reflux disease (GERD) are commonly addressed, rare conditions may only be encountered a few times or learned about through textbooks. Resident schedules require frequent transitions between inpatient and outpatient clinical settings, further adding to the complexity of primary care delivery and barriers to continuity. 

Insulinomas are rare pancreatic neuroendocrine tumors that secrete insulin, the primary hormone responsible for regulating blood glucose levels. The reported incidence of insulinomas ranges from 0.7 to four cases per million people per year [1-6]. The clinical presentation is classically characterized by Whipple's triad: signs or symptoms of hypoglycemia, measured hypoglycemia when these symptoms occur, and relief of symptoms upon normalization of blood glucose levels [7]. Meanwhile, approximately 38.4 million Americans (11.6% of the US population) have diabetes, with the most recent data from August 2021 to August 2023 showing a prevalence of among 15.8% among adults [8,9]. Among patients on insulin therapy for type 2 diabetes, 50% will experience mild to moderate hypoglycemia, and one in five will endure a severe episode of hypoglycemia [10]. This case report describes a patient with type 2 diabetes who developed an insulinoma, highlighting the challenges resident physicians face in diagnosing rare diseases within a continuity clinic framework.

This article was previously presented as a case report during the poster presentation session at the 41st Annual Multi-Campus Family Medicine Research Day in Los Angeles on May 14, 2025.

## Case presentation

A 67-year-old woman presented to a resident continuity clinic for a routine follow-up for chronic conditions, including management of diabetes. Her comorbidities included type 2 diabetes (diagnosed over 10 years ago), hypertension, hyperlipidemia, coronary artery disease status post-stent placement, depression, and GERD. She had been a patient in a resident-based primary care clinic since 2015, and throughout her care, she had poorly controlled diabetes requiring insulin therapy. In May 2018, her hemoglobin A1c (HgbA1c) was 9.8% despite being on 55 units of Neutral Protamine Hagedorn (NPH) insulin twice daily and 25 units of regular insulin twice daily. By May 2019, her HgbA1c decreased to 6.6%, remaining between 6% and 7% for subsequent years, despite the gradual reduction of her insulin dose and regimen. Eventually, this led to the complete discontinuation of her insulin regimen in 2020 and continuation only on oral medications such as metformin.

**Figure 1:**
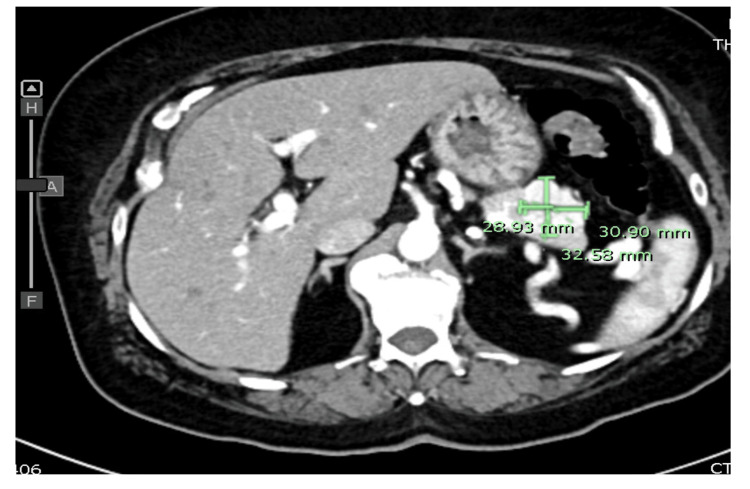
Computed tomography of the abdomen and pelvis with contrast Enhancing structure in the tail of the pancreas as described may be mildly increased in size. The appearance is suggestive of an intrapancreatic splenule.​

Her first episode of symptomatic hypoglycemia occurred in April 2022, when she reported sweating and shaking, with a blood glucose measurement of 64 mg/dL. Following this, she began reducing her metformin dosage on her own accord, reporting inconsistent use at follow-up visits over the next year. On a visit in April 2023, she exhibited presyncope, nausea, and weakness, with a blood glucose level of 54 mg/dL. In December 2023, her HgbA1c was 6.5%. In August 2024, she was seen for emergency room (ER) follow-up for dizziness and falls due to hypoglycemia, and her HgbA1c had dropped to 5.4% despite being off all diabetic medications. This prompted an order for a STAT computed tomography (CT) of the abdomen and pelvis with contrast, additional labs, and an Endocrinology referral. The diagnostic evaluation revealed elevated insulin (47.8 μU/mL; reference range: 2-25 μU/mL) and c-peptide (11.2 ng/mL; reference range: 0.5-2 ng/mL) in the presence of hypoglycemia (serum glucose 42 mg/dL). Her CT scan (Figure 1) showed an enhancing structure in the tail of the pancreas, which had previously been noted incidentally on a 2018 scan but was smaller at that time.

**Figure 2:**
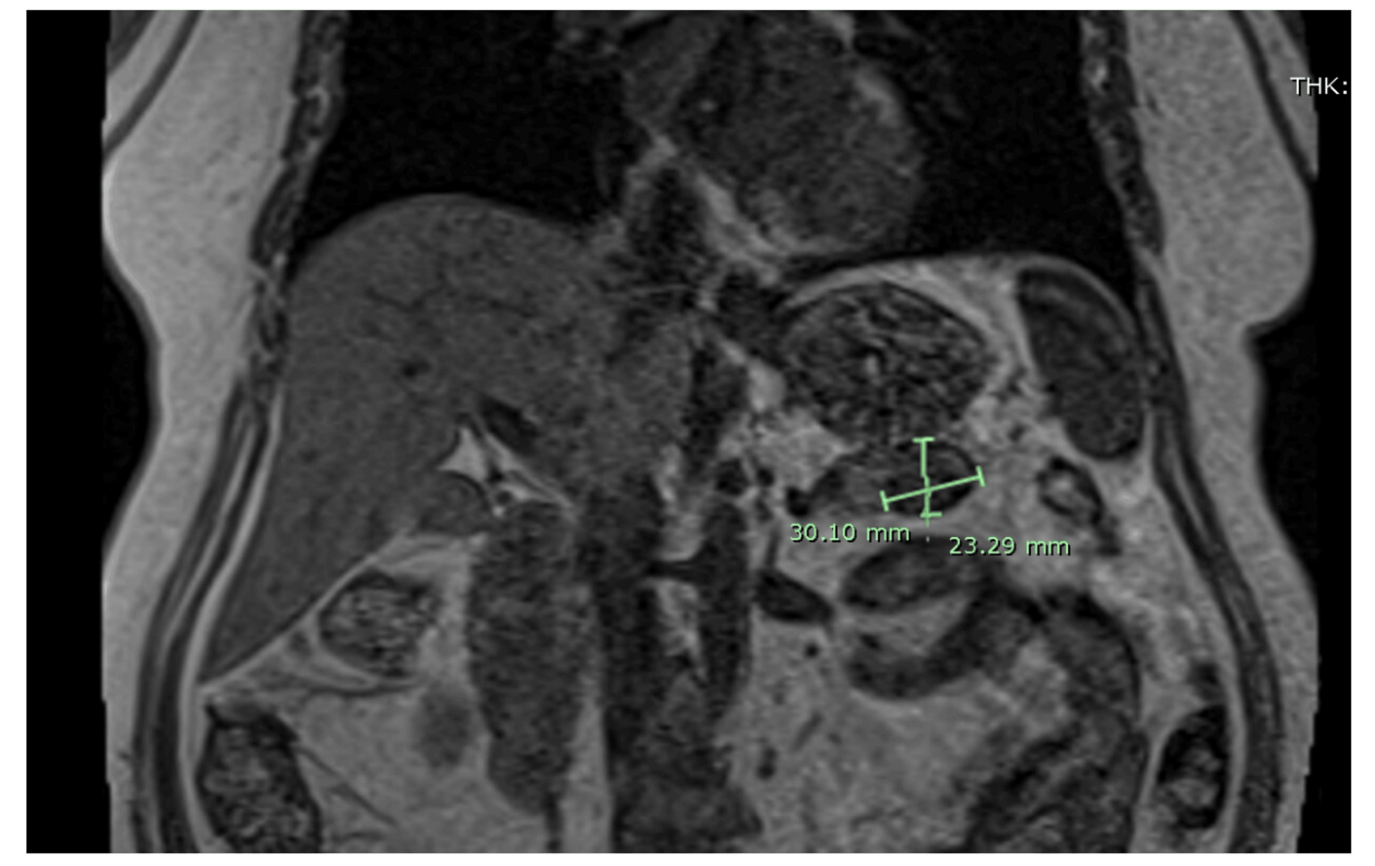
Magnetic resonance imaging of the abdomen and pelvis Pancreatic tail mass without significant enlargement since 2018 and demonstrating signal characteristics similar to the spleen is suggestive of an intrapancreatic splenule. No main pancreatic ductal dilatation. No suspicious lymphadenopathy.

A subsequent magnetic resonance imaging (MRI) (Figure 2) suggested an intrapancreatic splenule. The diagnosis of insulinoma was confirmed based on these findings, and she was referred to Surgical Oncology for removal.

In November 2024, she underwent distal pancreatectomy and splenectomy, with an uncomplicated postoperative course. She has remained a long-term patient in the resident continuity clinic and was seen by 10 different resident physicians between February 2022 and September 2024. Since her surgery, her HgbA1c has been poorly controlled, increasing to over 8%, and she has been restarted on insulin therapy with guidance from Endocrinology.

## Discussion

This case report illustrates how rare diagnoses, such as insulinoma, can go undetected in a resident continuity clinic. Outpatient care poses challenges, and diagnostic errors are not unique to trainees, with an estimated diagnostic error rate of around 5% per year, affecting approximately 12 million US adults [11]. However, resident clinics may be particularly susceptible to these errors due to factors like lack of continuity, balancing inpatient and outpatient duties, documentation lapses, and delays in diagnostic testing.

A 2016 case report highlighted delays in care that resulted in a patient with recurrent headaches failing to receive the correct Neurology referral after seeing three resident physicians in addition to her primary care provider [12]. This example underscores the complexities faced by residents in the outpatient setting, where they must juggle multiple tasks and unfamiliar patients.

Improved continuity has been shown to enhance patient outcomes. A systematic review found a strong correlation between continuity and improved diabetes outcomes in resident clinics, leading to greater resident satisfaction [13]. The Accreditation Council for Graduate Medical Education (ACGME) has responded with updated continuity requirements for Family Medicine residency programs, now mandating both individual resident visit and panel-wide data to assess this domain, with a target of 40% continuity for patient visits with assigned PGY3 primary care providers [14].

While continuity is a significant source of medical error in resident outpatient clinics, other challenges exist. A systematic review of diverse outpatient settings identified common sources of delayed diagnosis, including documentation fragmentation and failures in diagnostic follow-up [15]. To reduce diagnostic delays, residency programs must actively work to enhance outpatient clinical environments. The Association of American Medical Colleges (AAMC) provided guidelines in 2016 to structure resident primary care clinics, emphasizing active empanelment, continuity facilitation, interdisciplinary care coordination, and improved patient access to primary care providers [16]. Such changes can help mitigate diagnostic delays through systems-based interventions.

At an individual level, residents should maintain clinical curiosity and rely on thorough history-taking and physical examination to identify abnormal trends that may prompt the consideration of rare clinical diagnoses, such as insulinoma.

## Conclusions

This case report highlights how a rare diagnosis can be delayed in a primary care setting, particularly within a resident continuity clinic. Insulinoma is a rare cause of hypoglycemia and may be especially difficult to detect as insulin-dependent type 2 diabetes becomes increasingly prevalent in the United States. Notably, this case underscores that even "normal" or improving laboratory values, such as a declining HgbA1c, may represent underlying pathology rather than clinical improvement, emphasizing the need to interpret trends within the full clinical context.

Resident physicians face unique challenges that can contribute to diagnostic delays, with limited continuity of care being among the most significant. The ACGME has implemented important measures to address these barriers and improve care quality across Family Medicine residency programs. Ongoing evaluation of these changes and their impact on clinical outcomes will be essential.
